# miR-149 Alleviates Ox-LDL-Induced Endothelial Cell Injury by Promoting Autophagy through Akt/mTOR Pathway

**DOI:** 10.1155/2021/9963258

**Published:** 2021-08-26

**Authors:** Zhongsheng Zhu, Jinyu Li, Rui Tong, Xiaorong Zhang, Bo Yu

**Affiliations:** ^1^Department of Cardiology, Shanghai Pudong Hospital, Fudan University Pudong Medical Center, Pudong New District, Shanghai 201399, China; ^2^Department of Vascular Surgery, Shanghai Pudong Hospital, Fudan University Pudong Medical Center, Pudong New District, Shanghai 201399, China

## Abstract

**Background:**

Atherosclerosis is a chronic process that takes place in the vascular wall and causes various cardiovascular diseases (CVDs). Micro-RNA-149 (miR-149) mediates many physiological and pathological processes, including atherosclerosis. However, it is unclear about the roles of miR-149 in endothelial injury. Here, we explored the protective effect and related mechanism of miR-149 in endothelial cells induced with oxidized low-density lipoprotein (ox-LDL).

**Methods:**

Human endothelial cell lines (HUVECs) were exposed to ox-LDL to induce endothelial injury. Cell viability was determined by the CCK-8 assay. Autophagy was detected by immunofluorescence. RT-qPCR and western blot were carried out to determine the mRNA and protein expressions of Akt and mTOR.

**Results:**

The miR-149 level in HUVECs was reduced by ox-LDL (100 *μ*g/mL) incubation in a time-dependent manner. miR-149-mimic transfection markedly protected HUVECs from ox-LDL-induced injury, with increased cell viability and reduced caspase-3 activity. miR-149 mimics enhanced HUVEC autophagy, which was induced initially by ox-LDL. miR-149 mimics also markedly downregulated the expression of Akt, p-Akt, mTOR, and p-mTOR in ox-LDL-treated HUVECs. The miR-149-induced protection against HUVECs injury could be reversed by cotreatment with 3-methyladenine (3-MA, an autophagy inhibitor) or insulin (an activator of Akt/mTOR pathway).

**Conclusions:**

miR-149 prevents ox-LDL-induced endothelial cell injury by enhancing autophagy via increasing Akt and mTOR expressions.

## 1. Introduction

Atherosclerosis is a common chronic inflammatory disease of arterial endothelium, accumulating lipids and plaques in large arteries. Atherosclerosis can be initiated and developed from endothelial dysfunction, which has been regarded as an early predictor of atherosclerosis [1]. Various contributing factors could induce endothelial injury, among which, the most important is oxidized low-density lipoprotein (ox-LDL) [2]. Elevated serum levels of ox-LDL were found in all patients of atherosclerosis [3], and vascular endothelial cell injury was induced by ox-LDL through oxidative stress elevated levels [4]. Currently, it remains mostly unknown about the underlying complex molecular mechanism of atherosclerosis.

Autophagy is a metabolic process that degrades long-lived proteins and organelles during stress conditions and is essential for various physiological processes, such as cell growth and survival [5]. Accumulating data have indicated that autophagy plays a potential role in homeostasis and function of heart and vessel, and defective or excessive autophagy promotes atherosclerosis; however, the exact effect of autophagy on atherosclerosis is dependent on different cell types and stages of plaque [6]. Autophagy provides dual effects on cell injury in atherosclerosis. Therefore, the impact of autophagy in atherosclerosis still holds a need to be investigated.

Micro-RNAs (miRNAs) are noncoding small RNA molecules, and they mediate gene silencing by binding to 3′-untranslated region (3′-UTR). miRNAs play multiple roles in endothelial cells, including proliferation, adhesion, migration, and survival [7, 8], and are associated with atherosclerosis [9] and autophagy [10].

miR-149, a multifunctional miRNA, has many physiological and pathological roles. Polymorphism of miR-149 confers susceptibility to ischemic stroke, a condition highly related to atherosclerosis [11]. In addition, miR-149 could regulate apoptotic cells' clearance by macrophages, which inhibits inflammation and necrotic core formation in atherosclerotic lesions [12]. Moreover, miR-149 could inhibit apoptosis and enhance proliferation and autophagy of chondrocytes [13]. However, it remains unclear about the regulation of miR-149 and autophagic activity in endothelial cells. Therefore, this study aimed to investigate the role and mechanisms of miR-149 in endothelial cell injury induced with ox-LDL.

## 2. Materials and Methods

### 2.1. Cell Culture

HUVECs were purchased from the Cell Bank of Chinese Academy of Sciences (Shanghai, China) and cultured in low glucose Dulbecco's Modified Eagle Medium (DMEM) containing 10% FBS in an incubator with 5% CO_2_ at 37°C. HUVECs were exposed to ox-LDL (100 *μ*g/mL) to induce endothelial injury. To analyze the miR-149 expression after ox-LDL exposure, HUVECs were incubated with ox-LDL for 3, 6, 12, 24, and 48 h. HUVECs were divided into six groups to investigate cell injury: the control, NC mimics, miR-149 mimics, ox-LDL, ox-LDL + NC mimics, and ox-LDL + miR-149 mimics. HUVECs were split into three groups to investigate autophagy: the control, ox-LDL, and ox-LDL + miR-149 mimics. The control group was cultured with 0.1% dimethyl sulfoxide (DMSO).

### 2.2. Cell Transfection

HUVECs were seeded in 6-well plates in the DMEM medium with 10% fetal bovine serum (FBS). When cells grow to 70% confluence, they were transfected with miR-149 mimics or negative control (NC mimics) using lipofectamine 2000 reagent (Life Technologies, NY, USA). The mimic sequences (Sangon Biotech, Shanghai, China) were as follows: miR-149-5p mimics, 5′-UCU GGC UCC GUG UCU UCA CUC CC-3′; and mimics NC, 5′-UUC UCC GAA CGU GUC ACG UTT-3′. After transfection for 48 h, the transfection efficiency was confirmed by RT-qPCR and western blot.

### 2.3. Cell Viability Assay

HUVECs, HUVECs transfected with NC mimics, and HUVECs transfected with miR-149 mimics (all 5 × 10^3^ cells/well) were seeded in 96-well plates. Cells were then incubated with ox-LDL (100 *μ*g/mL) for 24 h. CCK-8 reagent was added to cells (1 : 10 dilution) for incubation at 37°C for 1 h. Absorbance was measured at a wavelength of 450 nm by a microplate reader (Bio-Rad, CA, USA).

### 2.4. Caspase-3 Activity

HUVECs were lysed and centrifuged to obtain the supernatants. Then, 30 *μ*L supernatants containing extracted protein were incubated with 70 *μ*L caspase-3 substrate (Ac-DEVD-pNA, cat. no.: C1115; Beyotime, Shanghai, China) at 37°C for 2 h. A microplate reader measured the absorbance at 405 nm. The caspase-3 activity was normalized to that of controls.

### 2.5. Immunofluorescence

HUVECs and HUVECs transfected with miR-149 mimics (3 × 10^4^/mL) were seeded in 6-well culture plates and were exposed to ox-LDL (100 *μ*g/mL) for 24 h. Cells were fixed with 4% paraformaldehyde (pH 7.4) for 20 min, permeabilized with 1% triton X-100, and blocked with 1% BSA. Cells were treated with goat polyclonal anti-LC3-II antibody (cat. no. sc-398822; Santa Cruz, CA, USA) at 4°C overnight and then incubated with FITC-linked secondary antibody for 1 h. Cells were stained with DAPI (10 mg/mL) and observed under a confocal microscope (Leica, Germany). The percentage of LC3-II + cells to DAPI + cells was calculated (at least 100 cells were counted).

### 2.6. Real-Time Quantitative PCR

Total RNA was extracted from HUVECs using TRIzol® reagent (Invitrogen). Template cDNA was synthesized from RNA by reverse transcriptase using ReverTra Ace qPCR RT kit (Toyobo, Tokyo, Japan). The qPCR reaction system was composed of cDNA (2 *μ*L), PCR master mix (10 *μ*L), forward primer (0.5 *μ*L), reverse primer (0.5 *μ*L), and H_2_O (7.5 *μ*L). The primer sequences were as follows: miR-149, forward 5′- GGC TCT GGC TCC GTG TCT T,′ reverse 5′- CAG TGC AGG GTC CGA GGT ATT-3′; U6, forward 5′-CTC GCT TCG GCA GCA CA-3′, and reverse 5′-AAC GCT TCA CGA ATT TGC GT-3′. All reactions ran the initial denaturation at 95°C for 7 minutes, followed by 40 cycles of denaturation at 95°C for 30 s and annealing at 60°C for 45 s. RT-qPCR was carried out using an ABI Prism 7500 Fast Real-time PCR instrument (Applied Biosystems; Foster City, CA, USA).

### 2.7. 2^−ΔΔCT^ Method

The miR-149 level was normalized to the expression of a small nuclear RNA (snRNA)-U6 and analyzed using the 2^−ΔΔCt^ method [14]. The 2^−ΔΔCt^ value was calculated according to the following formula: 2^(−ΔΔCt)^ = (Ct (miR − 149) − Ct (U6)) of all groups/(Ct(miR − 149) − Ct (U6)) of the control group [14, 15].

### 2.8. Western Blot

Total protein was extracted from HUVEC cells, followed by protein quantification using the bicinchoninic acid assay. The protein sample (50 *μ*g) was subjected to 10% SDS-PAGE electrophoresis and then transferred onto the PVDF membrane. To block the nonspecific protein binding site, the membrane was incubated with 5% low fat milk, treated with primary antibodies against Akt (cat. no. 4691S; Cell Signaling Technology, Inc.), p-Akt (cat. no. 4060), mTOR (cat. no. 2983), p-mTOR (cat. no. 5536), and *β*-actin at 4°C overnight. All primary antibodies were diluted to 1 : 500. Then, the blots were incubated with HRP-linked secondary antibody for 1 h. The bands were observed using enhanced chemiluminescence (ECL) (Thermo Scientific, Waltham, MA, USA), and their optical density was analyzed using ImageJ software.

### 2.9. Statistical Analysis

All quantitative values were expressed as means ± standard deviation (SD) and analyzed by SPSS 20.0 statistical software (SPSS Inc., Chicago, IL, USA). Multiple comparisons were achieved by one-way ANOVA, followed by Bonferroni's correction for post hoc testing. *P* < 0.05 was considered statistically significant.

## 3. Results

### 3.1. miR-149 Mimics Protected HUVECs from Ox-LDL-Induced Cell Injury and Apoptosis

**Figure 1:**
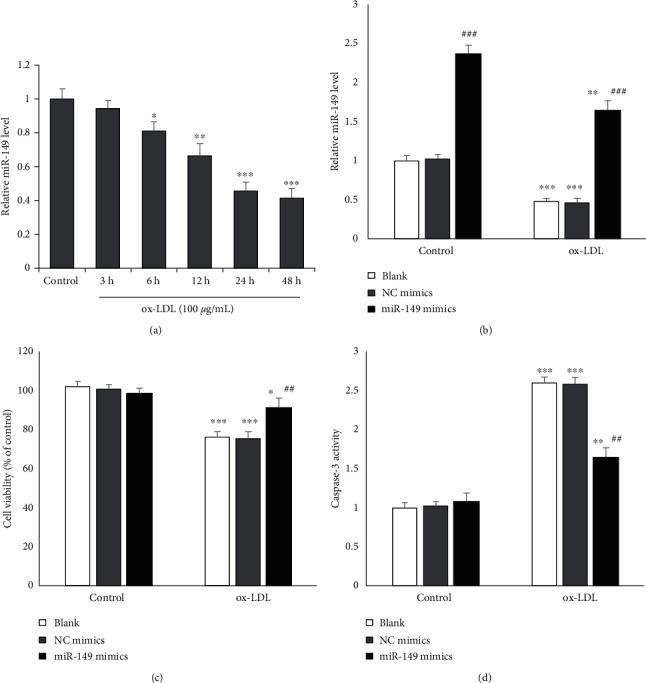
miR-149 mimics protect against HUVECs injury. (a) miR-49 level was reduced after ox-LDL incubation. (b) miR-49 level was increased after transfecting with miR-149 mimics for 24 h in HUVECs. (c) miR-149 mimics transfection increased cell viability. (d) miR-149 mimics transfection reduced caspase-3 activity.  ^*∗*^*P* < 0.05,  ^*∗∗*^*P* < 0.01, and  ^*∗∗∗*^*P* < 0.001 vs. control group; ^##^*P* < 0.01 vs. ox-LDL group.

HUVECs were incubated with ox-LDL (100 *μ*g/mL) for 3, 6, 12, 24, or 48 h to induce endothelial injury. RT-qPCR showed that miR-149 expression was markedly decreased by ox-LDL exposure in a time-dependent manner (Figure 1(a)), with a significant difference at all time points except 3 h. We then chose 24 h as the optimal incubation time for further experiments. To explore the effect of miR-149 on endothelial injury, HUVECs were transfected with miR-149 mimics or negative control (NC) mimics. miR-149 mimics transfection markedly increased the miR-149 level in cells with or without ox-LDL (Figure 1(b)). Moreover, miR-149 mimics markedly increased cell viability in HUVECs (Figure 1(c)). Furthermore, miR-149 mimics decrease caspase-3 activity in HUVECs with ox-LDL (100 mg/ml) (Figure 1(d)).

### 3.2. miR-149 Promoted Autophagy in HUVECs with Ox-LDL

**Figure 2:**
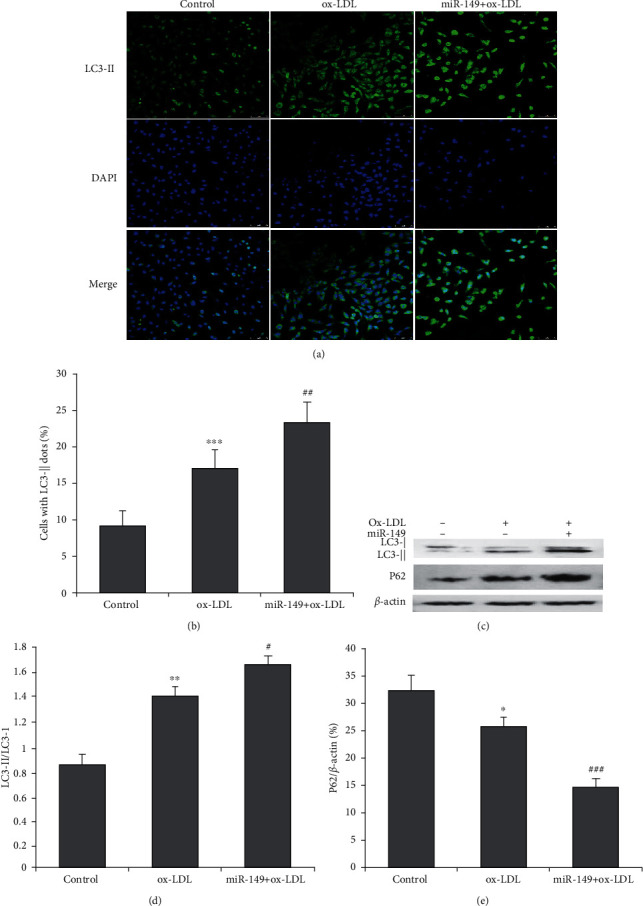
Effect of miR-149 mimics on autophagy in ox-LDL-induced HUVECs. (a) HUVECs of control, ox-LDL, and miR-149 mimics were stained with LC3-II antibody (green, magnification ×100). The nucleus was stained with DAPI (blue). (b) Quantification of LC3-II fluorescence intensity, as presented by cells with LC3-II dots. miR-149 mimics significantly increase the number of cells with LC3-II dots. (c) Western blot was performed to determine LC3-I, LC3-II, and P62 proteins. (d) miR-149 mimics significantly increases the ratio of LC3-II to LC3-I. (e) miR-149 mimics significantly decreases the P62 protein expression.  ^*∗∗∗*^*P* < 0.001 vs. control group; ^#^*P* < 0.05, ^##^*P* < 0.01, and ^###^*P* < 0.001 vs. ox-LDL group.

To assess whether miR-149 regulates autophagy, HUVECs were stained with LC3-II antibody and observed under confocal microscopy. Autophagosomes were represented by the yellow images, which were obtained from merging green puncta (FITC-linked LC3-II) with blue puncta (DAPI-stained nucleus). miR-149 mimics enhanced autophagy in HUVECs (yellow fluorescence) with ox-LDL induction (Figure 2(a)), with a significantly increased percentage of LC3-II + cells compared to cells with ox-LDL alone (Figure 2(b)). Furthermore, western blot showed that miR-149 mimics increased the LC3-II/LC3-I ratio and decreased P62 protein in HUVEC with ox-LDL (Figures 2(c)–2(e)).

### 3.3. miR-149 Modulated Akt/mTOR Pathway in HUVECs with Ox-LDL

**Figure 3:**
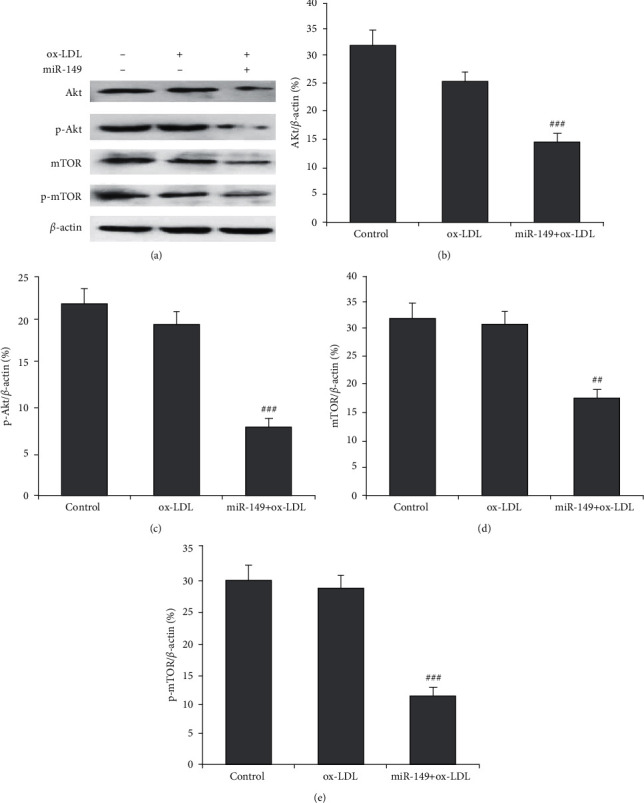
miR-149 mimics enhance autophagy through inhibiting the Akt/mTOR pathway. (a) Western blot was performed to determine Akt and mTOR proteins. Compared to HUVECs with ox-LDL (100 *μ*g/mL), expressions of Akt (b), p-Akt (c), mTOR (d), and p-mTOR (e) were significantly reduced by miR-149 mimics treatment. Calculation of all these proteins was normalized to *β*-actin. RT-qPCR was used to determine the mRNA expression of AKT.  ^*∗∗∗*^*P* < 0.001 vs. ox-LDL group.

We then explored whether miR-149 regulates the Akt/mTOR pathway, which involves the process of autophagy. Western blot was carried out to determine the protein expression of Akt, mTOR, and their phosphorylated forms (Figure 3(a)). miR-149 prominently repressed the total and phosphorylated Akt expressions (Figures 3(b) and 3(c)). Also, miR-149 reduced the total mTOR and the phosphorylated mTOR expressions (Figures 3(d) and 3(e)). Quantitative real-time PCR showed miR-149 reduced the mRNA expression of ATK in HUVEC with ox-LDL. The results indicated that suppression of the Akt/mTOR pathway might mediate enhanced autophagy by miR-149.

### 3.4. miR-149 Attenuated Ox-LDL-Induced Cell Injury by Enhancing Autophagy through Akt/mTOR Pathway

**Figure 4:**
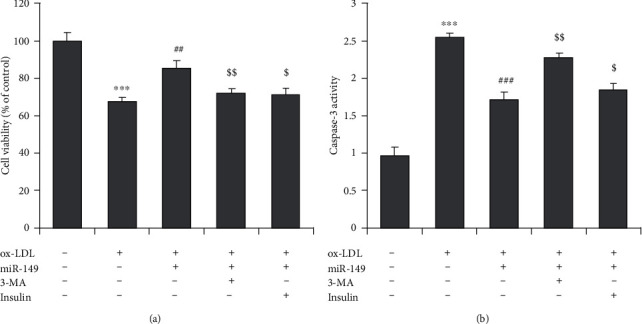
Enhanced autophagy and suppressed Akt/mTOR mediate the protective effects of miR-149 mimics. The inhibitor of autophagy (3-methyladenine, 3-MA, 100 *μ*m) and activator of Akt/mTOR pathway (insulin, 200 nm) were used. (a) Cell viability of HUVECs. (b) Caspase-3 activity of HUVECs.  ^*∗∗∗*^*P* < 0.001 vs. control group; ^##^*P* < 0.01 and ^###^*P* < 0.001 vs. ox-LDL group;  ^$^*P* < 0.05 and  ^$$^*P* < 0.01 vs. miR-149 group.

HUVECs were cotreated with 3-methyladenine (3-MA, an autophagy inhibitor) or insulin (an activator of the Akt/mTOR pathway). 3-MA or insulin cotreatment markedly reversed the miR-149-induced increase in cell viability (Figure 4(a)) and decrease in caspase-3 activity (Figure 4(b)).

## 4. Discussion

This study explored the function of miR-149 and its downstream pathway in endothelial injury. Our result suggests that miR-149 expression was declined in HUVECs by ox-LDL incubation. Moreover, miR-149 overexpression was found to increase cell viability and reduce caspase-3 activity in HUVECs induced by ox-LDL. Furthermore, miR-149-5 was confirmed to enhance autophagy and decrease expressions in Akt, p-Akt, mTOR, and p-mTOR in ox-LDL-treated HUVECs. The protective effects of miR-149 on HUVECs injury could be reversed by inhibition of autophagy or the Akt/mTOR pathway activation.

Ox-LDL mediates the initiation and progression of atherosclerosis by damaging endothelial cells [16]. Therefore, ox-LDL is an effective inducer of endothelial injury and can simulate initiation and development atherosclerosis, which is supported by our results that ox-LDL caused reduced HUVECs viability and increased apoptosis. The evidence suggests that HUVECs incubation with ox-LDL can simulate endothelial injury in atherosclerosis. Furthermore, ox-LDL reduced miR-149 level in HUVECs, and miR-149 overexpression prevented an ox-LDL-induced endothelial injury. A recent study got a similar result to ours that miR-149-5p attenuated ox-LDL-induced HUVECs injury [17]. Our results are also supported by a previous report that miR-149-5p promoted cell survival in high glucose-induced HUVECs. This protection was associated with a marked reduction in the levels of endothelin-1 (ET-1), von Willebrand factor (vWF), and ICAM-1 and an increase in the level of NO and eNOS [18]. ET-1, vWF, and ICAM-1 are all biomarkers of endothelial dysfunction and implicate atherosclerosis progression [19]. Conversely, eNOS can promote nitric oxide production (NO) and maintain endothelial homeostasis through sprouting of endothelial cells and repair of damaged endothelium [20]. This indicates that miR-149 is a protective molecule of endothelial injury in vascular diseases. miR-149-5p attenuated blood-brain barrier permeability and improved the outcomes of rats with cerebral ischemia [21]. In addition, miR-149-3p secreted by prostate cancer cells stimulated proliferation and motility of vascular endothelial cells [22]. Thus, because the miR-149 level was reduced by ox-LDL induction, our study added miR-149 as a new therapeutic agent for endothelial protection in atherosclerosis.

Autophagy is a conserved metabolic process with wide regulatory roles in the pathogenesis of atherosclerosis. Ox-LDL is reported to be an inducer of autophagy and in HUVECs [23, 24], which is consistent with our study, as evidenced by enhanced LC3-II immunofluorescence staining increased LC3-II/LC3-I ratio and decreased P62 protein expression in HUVECs. To date, several miRNAs, including miR-126 and miR-155, have been reported to promote autophagy in endothelial dysfunction induced by ox-LDL [25, 26]. However, the effect of miR-149 on autophagy of ox-LDL-induced HUVECs remains unclear. Our study showed that miR-149 mimics increased the percentage of LC3-II + cells in HUVECs with ox-LDL, and this result was supported by another report that miR-149 promoted autophagy in human primary chondrocytes [13]. Furthermore, our study showed that blockage of autophagy by 3-methyladenine (3-MA) reversed miR-149 mimics-induced suppression on ox-LDL-induced HUVECs injury. These results provide evidence that autophagic promotion by miR-149 has a beneficial effect on endothelial injury in atherosclerosis.

Akt/mTOR mediates a variety of cellular processes, such as apoptosis and autophagy [25]. It can suppress autophagy in macrophages and increase the vulnerability of atherosclerotic plaques [27]. Inhibition of Akt/mTOR also promoted autophagy in HUVECs with ox-LDL exposure [25, 28]. Our study showed that miR-149 overexpression reduced protein levels of Akt, p-Akt, mTOR, and p-mTOR in ox-LDL-induced HUVECs, which is consistent with the results in another report in human hepatocellular carcinoma (HCC) cells [28]. However, miR-126 reduced p-Akt and p-mTOR, but the total Akt and mTOR proteins remain unchanged [25]. This can be explained by the fact that there is a complementary site between the miRNA-149 seed region and 3′-UTR of the AKT1 mRNA. Thus, a luciferase reporter assay showed that miR-149 directly targeted Akt1 in HCC [29]. The direct targeting evidence of miR-149 to Akt in HUVECs deserves further investigation.

## 5. Conclusions

To sum up, miR-149 alleviates ox-LDL-induced endothelial injury by promoting autophagy via inhibiting the Akt/mTOR pathway. This study provides miR-149 as a potential target molecule for the prevention and therapy of atherosclerosis. The detailed process and mechanism modulated by miR-149 need further investigation, including in vivo experiments.

## Data Availability

The data in support of the results are available from the corresponding author upon reasonable request.
